# MiR-125a-3p-KLF15-BCAA Regulates the Skeletal Muscle Branched-Chain Amino Acid Metabolism in Nile Tilapia (*Oreochromis niloticus*) During Starvation

**DOI:** 10.3389/fgene.2020.00852

**Published:** 2020-08-11

**Authors:** Honghui Li, Xiaoling An, Lingsheng Bao, Yulong Li, Yaxiong Pan, Jinggang He, Li Liu, Xin Zhu, Jianshe Zhang, Jia Cheng, Wuying Chu

**Affiliations:** ^1^Hunan Provincial Key Laboratory of Nutrition and Quality Control of Aquatic Animals, College of Biological and Environmental Engineering, Changsha University, Changsha, China; ^2^Guangdong Provincial Key Laboratory of Food Quality and Safety, College of Food Science, South China Agricultural University, Guangzhou, China; ^3^Hunan Fisheries Science Institute, Changsha, China

**Keywords:** *Oreochromis niloticus*, miR-125a-3p-KLF15-BCAA, skeletal muscle, branched-chain amino acid metabolism, starvation

## Abstract

The branched-chain amino acids (BCAAs) play a key role in the energy metabolism of the muscle tissue and the Krüppel-like factor 15 (KLF15) as a transcription factor, which is a key regulator of BCAA metabolism in the skeletal muscle. This study assessed the effect of starvation for 0, 3, 7, and 15 days on BCAA metabolism in the skeletal muscle of Nile tilapia. The results showed that the expression of KLF15 showed a trend of increasing first and then decreasing during starvation, as well as the expression and activity of branched-chain aminotransferase 2 (BCAT2) and alanine aminotransferase (ALT). On the other hand, the content of BCAA was at first decreased and then upregulated, and it reached the lowest level after starvation for 3 days. In addition, through dual-luciferase reporter assay and injection experiments, it was found that KLF15 is the target gene of miR-125a-3p, which further verified that miR-125a-3p can regulate the BCAA metabolism by targeting KLF15 in the skeletal muscle. Thus, our work investigated the possible mechanisms of BCAA metabolism adapting to nutritional deficiency in the skeletal muscle of Nile tilapia and illustrated the regulation of BCAA metabolism through the miR-125a-3p-KLF15-BCAA pathway in the skeletal muscle.

## Introduction

Branched-chain amino acids (BCAAs) include leucine (Leu), isoleucine (Ile), and valine (Val). They are essential amino acids in humans and animals, accounting for about 35% of essential amino acids for muscle protein, which are important regulators of metabolism and metabolic health *in vivo*. The main metabolic site of BCAA is muscle (Yamane et al., 2017). Numerous studies have shown that the transcription factor of Krüppel-like factor (KLF) 15 (KLF15) is a key regulator of BCAA metabolism (Platell and Kong, 2001; Sun et al., 2016; Fan et al., 2018). The KLF15-BCAA signaling pathway is essential for metabolic homeostasis of the skeletal muscle. The BCAAs could not synthesize by themselves in the body. It must be obtained from food to satisfy the body’s needs. Most of amino acid metabolism in the body takes place in the liver, whereas BCAA is the only amino acid that is highly metabolized in the skeletal muscle (Brosnan and Brosnan, 2006). Studies have shown that the decomposition of BCAA is very active in the skeletal muscle. It can carry out transamination and complete oxidation at a fairly fast rate. The efficiency of ATP produced by complete oxidation of BCAA is much higher than that of other amino acids (Sperringer et al., 2017). When the body is in a resting state, about 14% of the total energy consumed by the skeletal muscle is provided by the process of BCAA oxidation. Under particular conditions such as starvation, exercise, and lactation, the increase in BCAA oxidation function is an important energy source *in vivo*. Studies have reported that the ability of muscle to oxidize α-ketoglutaric acid (α-KG), the transamination product of BCAA, can increase three to five times when deprived of food (Hsu et al., 2011; Wiśnik et al., 2011). However, starvation is a severe deficiency in caloric energy intake needed to maintain an organism’s life. Under fasting conditions, the pyruvate receives amino acids from glutamic acid generated by BCAAs in the skeletal muscle, which is converted into alanine by the transamination of alanine aminotransferase (ALT) and then transported to the liver. Pyruvate was generated by deamination and then converted into glucose, which provides gluconeous substrate for the liver to maintain energy metabolism and homeostasis balance (Felig, 1973; Perry et al., 2018).

The transcription factor of the KLF15 is a member of the KLF family. The family is characterized by three highly conserved transcriptional regulators of the DNA binding domains of the continuous Cys^2^/His^2^ zinc finger structure at the C-terminal, which play an important role in the growth and metabolism balance of the body. KLFs regulate the target gene expression and participate in cell growth, proliferation, differentiation, and apoptosis through binding specific GC-rich sequence in the promoter region of target genes, including GC box or GT box binding elements (Pearson et al., 2008; Leenders et al., 2012). At present, 18 members of KLFs have been identified; in particular, KLF15 has a high expression in organs with active metabolism such as the liver and skeletal muscle. This indicates its potential role in skeletal muscle metabolism (Uchida et al., 2000; Oates et al., 2001; Gray et al., 2002). Gray et al. (2007) found that after KLF15 gene knockout mice, the ability of skeletal muscle BCAA to generate alanine through transamination pathway was significantly reduced, and the expression level of branched-chain aminotransferase 2 (BCAT2) messenger RNA (mRNA), a key enzyme in the catabolism of BCAA, was significantly decreased. Moreover, the activity of ALT in the liver decreased significantly after KLF15 knockout. Shimizu et al. (2011) showed that KLF15 can regulate the expression of BCAT2 at the transcriptional level of rats. Jeyaraj et al. (2012) found in studies of normal mice and KLF15-deficient mice that overexpression of KLF15 adenovirus induces the mRNA level of ALT and that BCAT2 increased in the hepatocytes and muscle, and the content of glutamic acid (Glu) was increased and the alanine (Ala) decreased. This indicates that the abnormal expression of KLF15 mRNA level changes the metabolism of BCAAs. In addition, some studies have shown that the KLF15 has become an essential regulator in the metabolism of BCAAs, and this transcription factor deficiencies can cause serious damage to the organism (Morrison-Nozik et al., 2015; Fan et al., 2018).

MicroRNAs (miRNAs) are a family of endogenous, small, and non-coding RNAs that can negatively regulate gene expression at the post-transcriptional and/or translational level by binding loosely complementary sequences to 3′-UTR of the target gene (Bartel, 2004). MicroRNA-125a (miR-125a) is a vertebrate homolog of single-strand non-coding miRNAs (lin-4), the first miRNA reported in *Caenorhabditis elegans* (Lee et al., 1993). It has been found that miRNAs can regulate many important biological activities, including cell differentiation, proliferation, apoptosis, and metabolic homeostasis (Calin and Croce, 2006; Bousquet et al., 2012). Furthermore, miR-125a is also involved in regulating energy metabolism processes such as glucose metabolism, adipocyte differentiation, and amino acid metabolism (Kajimoto et al., 2006; Herrera et al., 2009; Sun et al., 2013). Some studies have reported the relationship between miRNA and glutamate metabolism in animals (Dang, 2009; Gao et al., 2009). The more active the cells are *in vivo*, the more active the glutamine metabolism was. Glutamine was converted into L-glutamic acid by glutamine enzyme and finally decomposed into ATP in mitochondria for energy supply or directly used as a substrate for glutathione synthesis. At present, the study of the miR-125a-3p-KLF15-BCAA signaling pathway on the metabolism of BCAAs in fish skeletal muscle has not been reported. In this study, we analyzed the regulation of the miR-125a-3p-KLF15-BCAA signaling pathway on the metabolism of BCAAs in the skeletal muscle of Nile tilapia by starvation experiment, which provided new ideas and basis for the regulation of muscle nutrition metabolism in Nile tilapia.

## Materials and Methods

### Animals and Experimental Design

The experimental fish were obtained from the National Tilapia Seed Farm (Nanning, China). A total of 180 healthy tilapias with an initial body weight of 199.86 ± 5.29 g were randomly assigned to four groups that fasted for 0, 3, 7, and 15 days with 3 replicate tanks per group and 15 fish in each tank after 2 weeks of domestication period. During the trial period, the optimal water temperature was kept at 27–30°C, and the experiment was carried out under natural light cycle. This experiment was approved by the Animal Care and Use Committee of Changsha University, and all animal experiments complied with the ARRIVE guidelines.

### Sample Collection and Amino Acid Analysis

At the end of the experimental period, fish in each tank were weighed and measured. After they were anesthetized with MS-222 (Green Hengxing Biotech Co., Ltd., Beijing, China), blood samples were collected from the caudal vein. After letting the samples stand, they were centrifuged at 3,500 *g* at 4°C for 15 min; the supernatant was then taken and stored at below ultralow temperature at −80°C for analysis. The collected skeletal muscle and other tissues were immediately put in liquid nitrogen and then stored at −80°C for analysis. The content of amino acids in the sample was determined by an automatic amino acid analyzer (Hitachi Model L8900).

### Enzyme Activity Analysis

The skeletal muscle sample was rinsed with 0.70% saline and homogenized on ice with 1:9 volume (v/w) saline. Then, the sample was centrifuged at 4,000 *g* for 15 min at 4°C, and the supernatant was collected for determination. According to the kit provided by Nanjing Jiancheng Bioengineering Research Institute of China, the total protein content and ALT activity of the skeletal muscle were measured. BCAT2 antibody was coated on a 96-well plate by ELISA, and its activity was measured. The optical density (OD) value was measured at 450 nm by an enzyme-labeling instrument, and the sample concentration was calculated.

### Quantitative Real-Time PCR Analysis

Total RNA was extracted using the TRIzol method. With the extracted RNA as a template, the first-strand cDNA was synthesized using the PrimeScript RT reagent Kit (with gDNA Eraser) (Takara, China). MiRNA reverse transcription was performed by the Mir-X miRNA First-Strand Synthesis Kit (Takara, China). According to our previous studies, using the above cDNA as the template, the quantitative real-time PCR (qRT-PCR) was performed for KLF15, BCAT2, ALT, miR-125a-3p, and β-actin (housekeeping gene) by the SYBR Premix Ex Taq II kit (Li et al., 2019). The primer sequences are indicated in Table 1. After the amplification efficiency of the primers was verified about 100%, the relative expression level of the tested genes was calculated by using the 2^–ΔΔCt^ method (Livak and Schmittgen, 2001).

**TABLE 1:** The primer sequence of real-time quantitative PCR.

Gene	Primer (5′–3′)	Annealing temperature (°C)
KLF15-F	5′-AAGAAGAGGAACGGTGCG-3′	58
KLF15-R	5′-TGGGATTTCTCGGGATTCTGT-3′	
BCAT2-F	5′-CGTCATCATCAGCCCAGTCG-3′	58
BCAT2-R	5′-CGTCATCATCAGCCCAGTCG-3′	
ALT-F	5′-AGCCAATCACTTTCTTCCGAC-3′	56
ALT-R	5′-TGTTCCCACCACAAGACTGC-3′	
MiR-125a-3p-F	5′-ACAGGTGAGGTTCTTGGGAG-3′	58
MRQ 3′primer	5′-CTCAACTGGTGTCGTGGAGTCGGCAATTCAGTTGAGC-3′	
β-Actin-F	5′-CCACAGCCGAGAGGGAAAT-3′	58
β-Actin-R	5′-CCATCTCCTGCTCGAAGTC-3′	

**mRQ 3’ primer was the general primer provided by TAKARA. KLF15, Krüppel-like factor 15; BCAT2, branched-chain aminotransferase 2; ALT, alanine transaminase.**

### Dual-Luciferase Reporter Gene Assay

In order to determine whether miR-125a-3p can directly recognize the 3′-UTR of KLF15 mRNA, we constructed the pGL4-CMV-luc-KLF15-WT and pGL4-CMV-luc-KLF15-Mut dual-luciferase reporter gene expression vectors. 293T cells were inoculated in a 24-well culture plate and cultured to 70–90% fusion. Cells were cotransfected with 20 μM of miR-125a-3p mimics or miR-NC, 5 ng plasmid of the KLF15-WT and KLF15-Mut and 10 ng reference plasmid of pRL-CMV, respectively. Luciferase activity was measured after transfection for 48 h by using a Dual-Luciferase Assay kit (Dual-Luciferase Reporter Assay System, Promega).

### Injection With LNA-125i in Nile Tilapia

A total of 36 fish with an initial body weight of about 200 g were randomly divided into control (injection with saline) and experimental (injection with LNA-125i) groups (6 fish in each group) fed with a commercial diet, which were obtained from the National Tilapia Seed Farm of Nanning.

Locked nucleic acid (LNA) is a novel oligonucleotide analog, and its bicyclic nucleotide derivative is locked in an RNA-mimicking sugar conformation, which can specifically inhibit miRNAs. The LNA-inhibitor-miR-125 (LNA-125i, Thermo Fisher) was dissolved in 0.65% normal saline (concentration of 0.25 mg/ml). The fish assigned to the experimental group received intraperitoneal injection of LNA-125i for a week (1 ml per fish every other day), and the control group was injected with the same volume of 0.65% normal saline.

### Statistical Analysis

Data were analyzed by one-way ANOVA and significance test with the SPSS 18.0 software. All data were expressed by mean ± SE, and Duncan’s multiple range tests were used to determine the significant difference between groups (*p* < 0.05).

## Results

### Expression of Starvation on Krüppel-Like Factor 15 and Branched-Chain Amino Acid Metabolism-Related Enzymes Messenger RNA Level in the Skeletal Muscle

**FIGURE 1:**
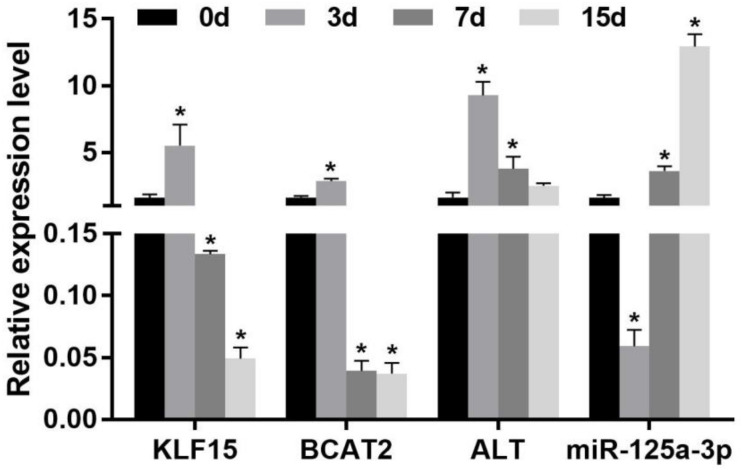
Effect of the expression of the miR-125a-3p-KLF15-BCAA- related gene mRNA level in the skeletal muscle of Nile tilapia during starvation periods. Values represent the mean ± SE (*n* = 9 fish, 3 fish were sampled for each tank). *p < 0.05 significantly different vs. 0 day. BCAA, branched-chain amino acid.

The effect of starvation on the expression of KLF15 and BCAA metabolism-related enzyme mRNA levels in the skeletal muscle of Nile tilapia is shown in Figure 1. Compared with that at 0 day of the starvation group, the expression of the KLF15 mRNA level was significantly increased in the skeletal muscle after 3 days of starvation (*p* < 0.05). Upon starvation for 7 to 15 days, it significantly decreased and was lower than that in normal feeding fish (*p* < 0.05). The expression of BCAA metabolism-related enzymes BCAT2 and ALT was significantly increased in the skeletal muscle after 3 days of starvation (*p* < 0.05). After starvation for 7 days, the levels of BCAT2 and ALT mRNA were significantly decreased; besides, the BCAT2 mRNA was significantly lower than that at starvation for 0 day (*p* < 0.05). After starvation for 15 days, the effects of ALT was decreased (*p* < 0.05), whereas BCAT2 displayed no significant change compared with 7 days of the starvation group (*p* > 0.05).

### Effect of Starvation on Branched-Chain Amino Acid Metabolism-Related Enzyme Activity in the Skeletal Muscle

**FIGURE 2:**
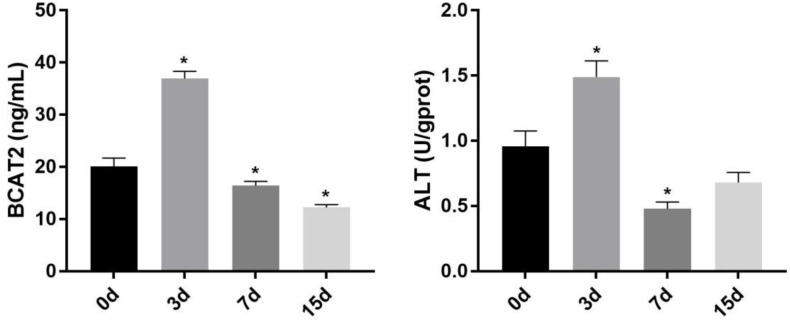
Effect of starvation on the activity of BCAT2 and ALT in the skeletal muscle of Nile tilapia. Values represent the mean ± SE (*n* = 9 fish, 3 fish were sampled for each tank). *p < 0.05 significantly different vs. 0 day. ALT, alanine aminotransferase.

The change of the activity of BCAA metabolism-related enzymes in the skeletal muscle of Nile tilapia during starvation is shown in Figure 2. The activity of BCAT2 and ALT was significantly increased in the skeletal muscle after starvation for 3 days (*p* < 0.05). However, after 7 days of starvation, their activity decreased remarkably, and both of them were lower than those of starvation for the 0-day group (*p* < 0.05). Compared with that after 7 days of starvation, the BCAT2 activity continued to decrease after 15 days of starvation, whereas ALT increased significantly (*p* < 0.05).

### Changes of Branched-Chain Amino Acid Content in the Skeletal Muscle

The change of the BCAA content in the skeletal muscle of Nile tilapia under starvation periods is shown in Table 2. The results showed that the content of BCAA was decreased significantly in the skeletal muscle after starvation for 3–7 days and reached the lowest level after starvation for 3 days (*p* < 0.05). Then, the BCAA content was increased significantly after 15 days of starvation and returned to starvation 0-day level (*p* > 0.05).

**TABLE 2:** Effect of starvation on BCAA content in the skeletal muscle of Nile tilapia (g/100 g).

	0 day	3 days	7 days	10 days
Leu	1.62 ± 0.13	1.45 ± 0.06	0.98 ± 0.06*	1.63 ± 0.08
Ile	0.87 ± 0.03	0.53 ± 0.02*	0.78 ± 0.03*	0.85 ± 0.05
Val	0.92 ± 0.04	0.56 ± 0.02*	0.86 ± 0.03	0.87 ± 0.05
BCAA	3.41 ± 0.20	2.54 ± 0.10*	2.62 ± 0.12*	3.35 ± 0.13

*Values represent the mean ± SE (n = 9 fish, 3 fish were sampled for each tank).* *p < 0.05 significantly different vs. 0 day. BCAA, branched-chain amino acid.

### Krüppel-Like Factor 15 Is a Direct Target of miR-125a-3p

**FIGURE 3:**
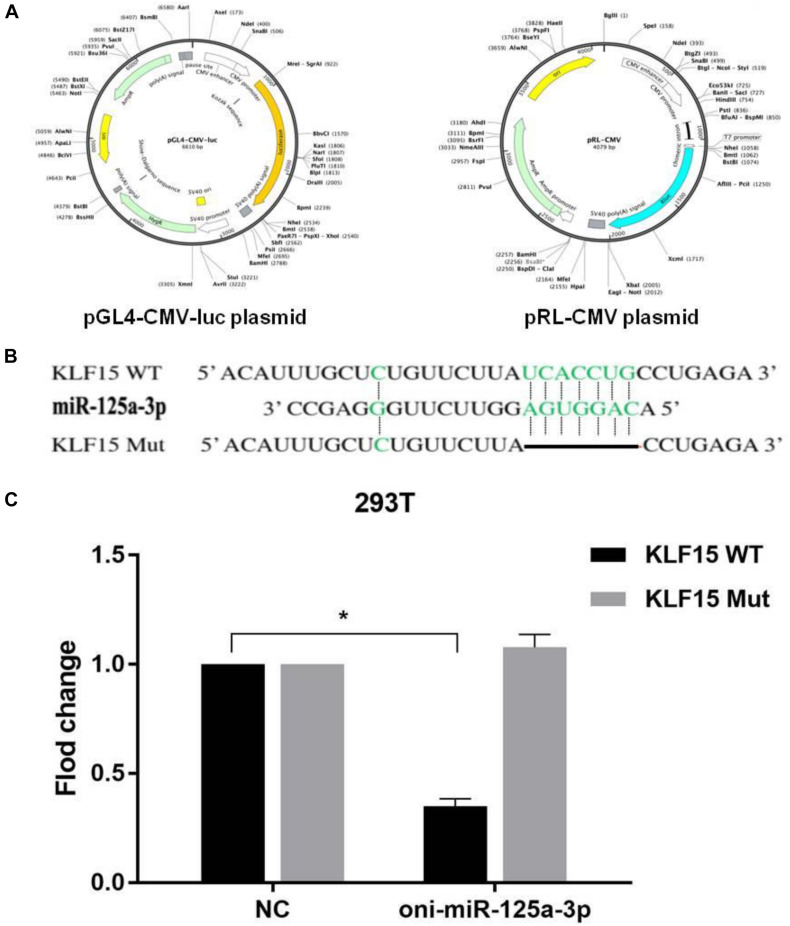
Results of dual-luciferase reporter gene assay. **(A)** The reporter plasmid of pGL4-CMV-luc and pRL-CMV. **(B)** miR-125a-3p and KLF15 3′-UTR have matching binding sites. **(C)** The interaction between miR-125a-3p with the KLF15 3′-UTR. *p < 0.05 significantly different vs. NC.

The target gene of miR-125a-3p was predicated by the TargetScan online program and verified using a luciferase reporter gene assay, and the reporter plasmid of pGL4-CMV-luc and pRL-CMV is shown in Figure 3A. The results showed that there was a binding site between miR-125a-3p and KLF15 3′-UTR (Figure 3B). Compared with that of the NC group, the fluorescence activity of KLF15-WT cells was significantly decreased in the miR-125a-3p group, whereas KLF15-Mut had no significant change (Figure 3C). This indicates that miR-125a-3p has a negative regulatory role in the KLF15 gene expression; therefore, KLF15 is a target gene of miR-125a-3p.

### Expression of Injection With LNA-125i on Krüppel-Like Factor 15 and Branched-Chain Amino Acid Metabolism-Related Enzymes Messenger RNA Level in the Skeletal Muscle

**FIGURE 4:**
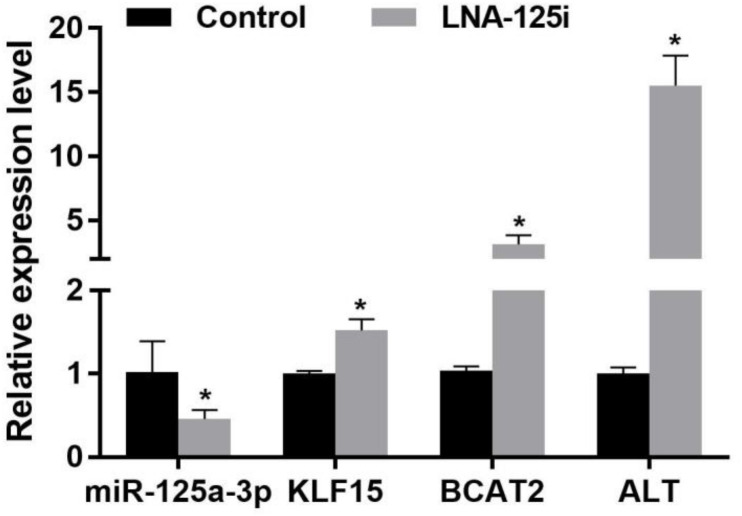
Effect of the expression of the miR-125a-3p-KLF15-BCAA- related gene mRNA level in the skeletal muscle of Nile tilapia after injection with LNA-125i. Values represent the mean ± SE (*n* = 9 fish, 3 fish were sampled for each tank). *p < 0.05 significantly different vs. control. BCAA, branched-chain amino acid.

The effect of LNA-125i injection on the expression of KLF15 and BCAA metabolism-related enzyme mRNA level in the skeletal muscle of Nile tilapia is shown in Figure 4. Compared with the control, LNA-125i injection significantly inhibited the expression of miR-125a-3p and enhanced the KLF15 mRNA level in the skeletal muscle (*p* < 0.05). After injection, the expression of BCAT2 and ALT was increased significantly compared with that of the control (*p* < 0.05).

### Effect of Injection With LNA-125i on Branched-Chain Amino Acid Metabolism-Related Enzyme Activity in the Skeletal Muscle

**FIGURE 5:**
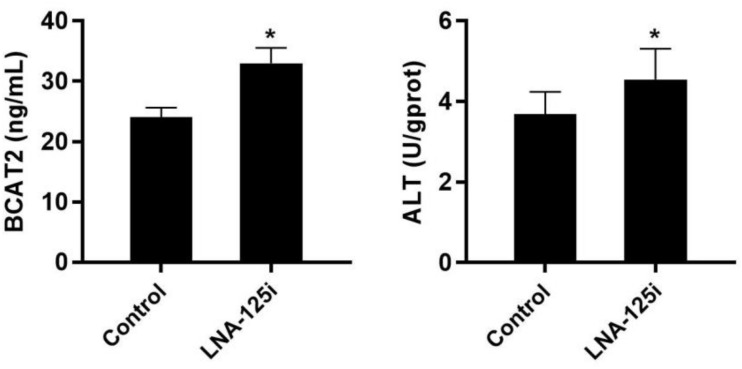
Effect of injection with LNA-125i on the activity of BCAT2 and ALT in the skeletal muscle of Nile tilapia. Values represent the mean ± SE (*n* = 9 fish, 3 fish were sampled for each tank). *p < 0.05 significantly different vs. control. ALT, alanine aminotransferase.

The change of BCAA metabolism-related enzyme activities after LNA-125i injection in the skeletal muscle of Nile tilapia is shown in Figure 5. Compared with that of the control, the activity of BCAT2 and ALT was increased significantly in the skeletal muscle after LNA-125i injection (*p* < 0.05).

### Changes of Injection With LNA-125i on Branched-Chain Amino Acid Content in the Skeletal Muscle

The change of the BCAA content treated with LNA-125i in the skeletal muscle of Nile tilapia is shown in Table 3. The results showed that the BCAA content was decreased significantly in the skeletal muscle after injection with LNA-125i (*p* < 0.05).

**TABLE 3:** Effect of injection with LNA-125i on BCAA content in the skeletal muscle of Nile tilapia (g/100 g).

	Control	LNA-125i
Leu	1.55 ± 0.03	1.38 ± 0.04*
Ile	0.69 ± 0.02	0.56 ± 0.02*
Val	1.19 ± 0.02	0.88 ± 0.03*
BCAA	3.43 ± 0.05	2.82 ± 0.07*

Values represent the mean ± SE (n = 9 fish, 3 fish were sampled for each tank). *p < 0.05 significantly different vs. control. BCAA, branched-chain amino acid.

## Discussion

The skeletal muscle is involved in many complete processes of the body. It is not only the main protein storage in the body but also conducts an active and adaptive metabolism that produces highly plastic tissue. It plays an important role in the energy metabolism of the body and is mobilized as an amino acid source of energy metabolism under stress (Lecker and Goldberg, 2002). Some studies have shown that the BCAA metabolism is mainly through intramuscular transamination, which is the primary way of endogenous synthesis of Glu, glutamine, and aspartic acid (Asp) (She et al., 2007; She et al., 2010). When the body is in a resting state, about 14% of the total energy consumed by the skeletal muscle was provided by the process of BCAA oxidation. Under particular conditions including starvation, exercise, and lactation, the increase in the BCAA oxidation function is an important energy source *in vivo* (Xu et al., 2009; Leenders et al., 2012; Prosdocimo et al., 2014). KLF15 plays a key role in many biological processes, which includes cell proliferation, differentiation, development, and apoptosis. Studies have confirmed that KLF15 was involved in the regulation of glucose metabolism, fatty acid metabolism, and amino acid metabolism. The study of KLF15 on the amino acid metabolism, especially on BCAA metabolism, was a new kind of research field. Our results showed that the expression of KLF15 mRNA was upregulated in the skeletal muscle of Nile tilapia after starvation for 3 days, whereas the gene expression and activities of BCAT2 and ALT increased significantly. Meanwhile, the content of BCAA decreased significantly. This may be due to the upregulation of KLF15, which stimulates the expression and activity of BCAT2 and ALT, thus promoting the catabolism of BCAA and reducing the contents. It has been reported that the overexpression of KLF15 could increase the activity of the BCAT2 promoter in the skeletal muscle and cardiomyocytes, and the content of BCAA was decreased in cells (Yoshikawa et al., 2009; Shimizu et al., 2011), which was consistent with our results. These results showed that the BCAAs in the skeletal muscle are used to maintain energy stability in Nile tilapia under starvation. Some studies have also found that catabolism of BCAAs needs to provide a carbon matrix for liver gluconeogenesis and then maintain normal blood glucose of the body during starvation (Gray et al., 2007). Additionally, KLF15 can inhibit the lipogenesis and promote gluconeogenesis by upregulating the key enzymes of BCAA decomposition at fasting state, and thus, it provides the liver with gluconeogenesis substrate (Teshigawara et al., 2005).

Then, the expression of KLF15 mRNA was downregulated in the skeletal muscle of Nile tilapia after starvation for 7 to 15 days, whereas the gene expression and activity of BCAT2 and ALT were both decreased significantly. However, the content of BCAA began to increase after 7 days of starvation and returned to starvation 0-day level at 15 days of starvation. This may be due to the fact that BCAA increases its own decomposition and provides energy for the body during starvation. In the process of starvation, the pyruvate receives amino acids from glutamic acid generated by BCAAs in the skeletal muscle, which is converted into alanine by the transamination of ALT and then transported to the liver. Pyruvate was generated by deamination and then converted into glucose, which provides gluconeous substrate for the liver to maintain energy metabolism and homeostasis balance. Perhaps after starvation, the BCAA increases its decomposition and provides energy for the body during starvation. However, when it reached a certain level, BCAA could not be decomposed in the skeletal muscle and gradually returned to the balance level. This might be due to the fact that KLF15 was involved in regulating several metabolic pathways *in vivo* during starvation and promoting the gluconeogenesis and provided the energy for maintaining life activities (Takeuchi et al., 2016). In addition, protein metabolism in the intestine and the liver was increased after long-term starvation, and the intake level of gluconeogenesis precursor in the liver also increased, which indicated that gluconeogenesis was increased (de Blaauw et al., 1996). The changes of BCAA metabolism in muscle after starvation might reflect the adaptive response of muscle to nutritional deficiency.

Among the most important miRNA families, miR-125 is a highly conserved miRNA throughout diverse species, and it has been validated to change, exhibiting its different roles in many different types of diseases (Sun et al., 2013). The MiR-125 family is composed of miR-125a-3p, miR-125a-5p, miR-125b-1, and miR-125b-2, which are highly conserved in evolution. Members of this family play an important role in many biological processes in cells, including proliferation, differentiation, apoptosis, and metabolism by targeting a variety of different transcription factors (Bousquet et al., 2012), matrix-metalloprotease (Qian et al., 2012; Xu et al., 2012), growth factors (Ge et al., 2011), and so on. The purpose of this study was to investigate the mechanism of the miR-125a-3p-KLF15-BCAA signaling pathway on BCAA metabolism in the skeletal muscle of Nile tilapia by *in vivo* experiments. It was found that KLF15 is the target gene of miR-125a-3p, and the negative regulation effect of miR-125a-3p on KLF15 was verified after LNA-125i injection. The miR-125a-3p could regulate the BCAA metabolism in the skeletal muscle of Nile tilapia by targeting the KLF15 gene. This study demonstrates that the miR-125a-3p-KLF15-BCAA signaling pathway is essential for the regulation of BCAA metabolism in the skeletal muscle of Nile tilapia.

In conclusion, the present results suggest that the miR-125a-3p-KLF15-BCAA signaling pathway plays a key role in the regulation of BCAA metabolism adaptive in the skeletal muscle of Nile tilapia under nutrition changes. In order to adapt to the change, the tilapia could activate the catabolism of BCAAs in the skeletal muscle to provide energy by the miR-125a-3p-KLF15-BCAA signaling pathway. These results provided a new idea for the regulation of muscle nutrition metabolism in Nile tilapia.

## Data Availability Statement

All datasets generated for this study are included in the article/supplementary material.

## Ethics Statement

The animal study was reviewed and approved by the Animal Care and Use Committee of Changsha University.

## Author Contributions

WC and JC carried out the conceptualization and performed the project administration. HL and XA performed the formal analysis and investigation. WC and JZ acquired the funding. HL, XA, YL, YP, JH, LL, and XZ carried out the methodology. WC performed the supervision. HL wrote the original draft. HL and WC reviewed and edited the manuscript. All authors contributed to the article and approved the submitted version.

## Conflict of Interest

The authors declare that the research was conducted in the absence of any commercial or financial relationships that could be construed as a potential conflict of interest.
